# CanWalk: study protocol for a randomized feasibility trial of a walking intervention for people with recurrent or metastatic cancer

**DOI:** 10.1186/s40814-015-0003-5

**Published:** 2015-03-12

**Authors:** Jenny Harris, Vicki Tsianakas, Emma Ream, Mieke Van Hemelrijck, Arnie Purushotham, Lorelei Mucci, James SA Green, Karen Robb, Jacquetta Fewster, Jo Armes

**Affiliations:** 1 Kings College London, Kings Health Partners, Florence Nightingale Faculty of Nursing and Midwifery, London, UK; 2 University of Surrey, School of Health Sciences, Faculty of Health and Medical Sciences, Guildford, UK; 3 King’s College London, Kings Health Partners, Division of Cancer Studies, London, UK; 4 Department of Epidemiology, Harvard School of Public Health, Boston, USA; 5 Department of Urology and North London East Regional Urological Cancer Centre, Whipps Cross Hospital, London, UK; 6 Department of Health and Social Care, London South Bank University, London, UK; 7 NHS England, London, UK; 8 Macmillan Cancer Support, London, UK

**Keywords:** Randomized controlled trial, Feasibility studies, Qualitative evaluation, Metastatic cancer, Recurrent cancer, Secondary cancer, Walking

## Abstract

**Background:**

Increasing numbers of people in the UK are living with recurrent or metastatic cancer, many of whom experience reduced quality of life resulting from the physical and psychosocial consequences of cancer and its treatment. While drug treatments are important at alleviating some symptoms, there is increasing evidence of the benefits of exercise in enhancing quality of life and health outcomes. Walking is an inexpensive and accessible form of exercise. To our knowledge, no studies have investigated whether a walking intervention is sufficient to enhance quality of life and alleviate symptoms in people with recurrent or metastatic cancer across a range of tumor types. This paper describes the CanWalk study protocol, which aims to assess the feasibility and acceptability of undertaking a randomized controlled trial of a community-based walking program to enhance quality of life and well-being in people with recurrent or metastatic cancer.

**Methods:**

A mixed methods feasibility study includes an exploratory two-center randomized controlled trial and qualitative interviews. A minimum of 60 participants will be recruited from two London NHS Trusts and randomized 1:1 between the walking intervention and standard care using minimization. The walking intervention consists of the initial provision of written/online information followed by a short motivational interview. Participants are instructed to walk for 30 min on alternate days and attend an organized volunteer-led walk once a week. Half of all participants will be asked to use a pedometer. Postal questionnaires will be completed at baseline (pre-randomization) and at 6, 12 and 24 weeks. A subsample of participants and stakeholders will be interviewed at the end of the study.

**Results:**

Primary outcomes will be the acceptability and feasibility of the intervention and trial. A range of secondary outcome assessments needed to design a main study, including estimates of recruitment, adherence and variability in quality of life, will be evaluated.

**Conclusions:**

Data from this study will be used to refine the walking intervention, investigate the acceptability of the intervention and study design, and determine the most appropriate outcome measures thereby providing estimates of the factors needed to design the main study.

**Trial registration:**

ISRCTN42072606.

## Background

Two million people are living with cancer in the UK today [1], and it is predicted that the number will increase to four million over the next 30 years [2]. The precise number of people living with recurrent cancer (the cancer has returned after treatment has ended) or metastatic cancer (the cancer has spread from the place where it first started) is unknown, although there is some evidence that the life expectancy of this group is also increasing [3]. Nevertheless, people with recurrent or metastatic cancer face a number of health challenges including psychological disorders [4] and physical symptoms, including pain, fatigue, and appetite loss [5]. While drug treatments are important at alleviating some of these symptoms, two recent systematic reviews identified the important potential contribution of exercise in enhancing the quality of life (QoL) in those with recurrent or metastatic cancer [6,7]. Specifically, programs that include regular walking for more than 30 min may generate improvements in QoL, physical functioning, and fatigue and are acceptable and well tolerated [6,8]. However, many of the interventions reviewed included different types of physical activity, were supervised, and required attendance at specialist exercise facilities, which may limit their acceptability or economic sustainability in the long term.

While many people with cancer may be hesitant to exercise [9], research from primary care demonstrates the effectiveness of brief advice, supported by written materials, at increasing physical activity levels [10]. Walking is an inexpensive form of exercise, it can be undertaken alone or in a group, and has demonstrated significant health benefits - including for cancer [11]. An additional advantage of walking is that it is not restricted to a specific facility or setting and has been shown to be associated with longer-term changes in behavior [10]. Preliminary results from a randomized pilot walking intervention in Sweden among men with prostate cancer suggest that regular group walking has positive effects on both quality of life and inflammatory and metabolic biomarkers [12]. However, no studies to date have investigated whether a walking intervention on its own is sufficient to enhance the physical and psychological well-being of people with recurrent or metastatic cancer across a range of tumor types.

Here, we describe the protocol of the CanWalk study, which aims to assess the feasibility and acceptability of undertaking a randomized controlled trial of a community-based walking program to enhance quality of life in people with recurrent or metastatic cancer. The study will promote a walking intervention, assess whether participants find it acceptable and evaluate whether a full randomized controlled trial is warranted and feasible. Specific objectives include:

1. The development of the walking intervention to encourage intervention uptake [13];
2. To investigate the acceptability to participants of a) the walking intervention, b) the study materials, and c) being randomized to intervention or control; and to assess d) the acceptability and timing of the selected outcome measures, and e) use of pedometers to enhance and measure adherence;
3. To provide estimates of key aspects of trial design needed to design a full-scale randomized trial design, if warranted, including; a) number of eligible participants, b) recruitment rate, c) retention rate, d) response rates to initial and follow up questionnaires, e) the utility of objective and subjective methods to assess adherence to the walking intervention, and 4) generation of an estimate of the variability in quality of life.

## Methods

### Design

This is a mixed methods [14] feasibility study based on an exploratory two-center randomized controlled trial (RCT) with nested qualitative interviews. The study is registered with the International Standard Randomized Control Trial Number Register (ISRCTN42072606). Participants will be randomized, using a central online system, 1:1 between the walking intervention and standard care using minimization (based on age, sex, and physical activity levels).

### Setting and participants

The study will be undertaken in two large London NHS Foundation Trusts. Participants are eligible for the study if they i) are 16 years or over, ii) have a diagnosis of breast, colorectal, upper gastrointestinal, gynecological, hematological, head and neck, melanoma, or prostate cancer with recurrent or metastatic disease, iii) meet the eligibility criteria for specific cancer diagnoses (Table 1), and iv) are able to walk for a minimum of 30 min unaided. Exclusion criteria are i) having bone metastases which the responsible health-care professional considers a contraindication to participating in the walking intervention; and ii) unable to speak and understand English.

**Table 1:** Walking intervention for people with recurrent or metastatic cancer: definitions of recurrent or metastatic disease

Primary tumor	Definition
Breast	Metastatic disease - stage 4, for example, visceral, bone, soft tissue, and so on.
	Not local recurrence
Colorectal	Metastatic - stage 4 - M1
Gynecological	Ovary - symptomatic stage 3/4/recurrence
	Cervix - stage 3/4
	Endometrial - stage 3/4
	Vulva - stage 3/4
Hematological	Lymphoma (high and low grade) within first 6 months of relapse, myeloma
Head and neck	Metastatic disease, recurrence
Melanoma	Stage 3/4
Prostate	Distant metastatic disease or bone metastases (T3, N1, M1)
Upper gastrointestinal	Stage 3/4, oesophago-gastric

**Figure 1:**
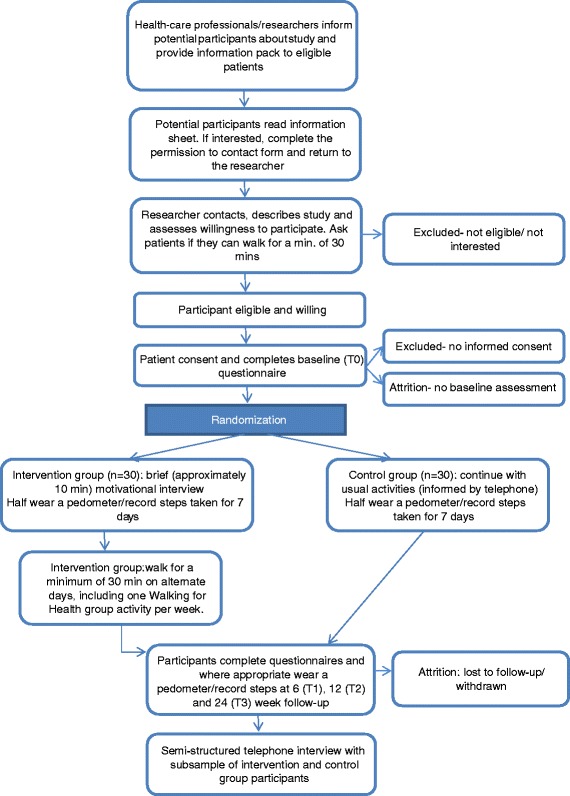
Walking intervention for people with recurrent or metastatic cancer: flowchart of study protocol.

Figure 1 shows the flow of participants through the study. Participants will be screened for eligibility by their health-care professional (that is, nurses or doctors) as they visit clinics or via hospital records. Health-care professionals or researchers will describe the study to those identified during a clinic attendance and where appropriate provide a study information pack comprising an invitation letter, information sheet, permission to contact form, a unique study identification number, and postage paid envelope. Those identified via hospital records will be sent the study information pack by post by their health-care professionals. Health-care professionals/researchers will record reasons for ineligibility and reasons for declining participation.

Patients who are interested in participating in the study will be asked to send their permission to contact form directly to the study researcher, giving consent for the researcher to make telephone contact to describe the study further, assess their willingness to participate, and check whether they can walk for a minimum of 30 min. Those who can, and are still interested in the study, will be sent a consent form, general practitioner (GP) form (to ascertain the details of their family doctor and permission for the researchers to write to inform their GP of their participation in the study), and a baseline questionnaire (T0). Possible outcomes are (1) eligible and willing to participate, (2) eligible and unwilling to participate, and (3) ineligible. Again, wherever possible, reasons for declining participation and ineligibility will be recorded.

### The intervention

The intervention comprises a brief (approximately 10-min) telephone or face-to-face session, based on the UK’s National Institute for Health and Clinical Excellence (NICE) guidance on promoting physical activity in primary care [13]. The study will utilize the preexisting Walking for Health program, a network of UK-wide, freely available walking groups provided by MacMillan Cancer Support and The Ramblers [15]. Participants in the intervention group will be asked to participate in at least one Walking for Health group activity per week and undertake walking for at least 30 min on alternate days over 3 months, either independently or with Walking for Health groups. Walking for Health’s volunteers lead more than 3,000 short walks (lasting approximately 30 min to 1 h) throughout the UK every week. The researcher will assess the patient’s readiness to adhere to the walking intervention and, using motivational interviewing techniques, stimulate their use of study materials as a means to increase physical activity. Increasingly used in health-care settings, motivational interviewing is a patient-centered, counseling style that augments an individual’s motivation to change behavior and move toward a specific goal [16]. It explores the person’s own reasons for change and focuses on their strengths, not just the obstacles and weaknesses associated with change [13,16]. Study materials will be provided in print and online formats to reinforce the intervention. Information on local Walking for Health groups will be provided, including details of the seven Walking for Health coordinators in South East London and how to find out about walks elsewhere. The intervention will be delivered by researchers trained in motivational interviewing. An expert in motivational interviewing will provide supervision to the researchers during this process to ensure adherence to operational procedures and the principles of motivational interviewing. Using random selection, intervention sessions will, with consent, be audio recorded to ensure fidelity to the intervention manual.

### Comparator group

Participants in the comparator group will be asked to continue their activities as usual during the study (standard care).

### Assessments

Participants will complete postal questionnaires at baseline (T0) and at 6 (T1), 12 (T2), and 24 (T3) weeks following recruitment to the study. Two postal reminders will be sent to non-responders. Table 2 provides an overview of the questionnaires that will be completed at each assessment with a rationale for their use. Additionally, those in the intervention group will be asked to record their Walking for Health participation - including date and location of walks attended - on a simple form. Semi-structured telephone interviews will be conducted with ten participants (five per study group) to assess the acceptability of the intervention, randomization, and evaluation of the study methods. At the end of the intervention, a maximum of ten stakeholders (that is, community group coordinators/walk leaders, clinical nurse specialists, research nurses and hospital doctors) will be interviewed by the study researchers (JH, VT) to explore the acceptability of the walking intervention from a professional perspective.

**Table 2:** Walking intervention for people with recurrent or metastatic cancer: questionnaire measures by assessment point

Measures included in the study questionnaire	Assessment point			
*Description*	T0 (Baseline)	T1 (6 weeks)^1^	T2 (12 weeks)^1^	T3 (24 weeks)^1^
* Functional assessment of cancer therapy- general* (*FACT-G*) [18]	✓	✓	✓	✓
• *Assesses emotional and physical quality of life in the past 7 days*				
* Depression, anxiety, stress scales* - *21* (*DASS-21*) [19]	✓	✓	✓	✓
• Assesses the frequency and severity of symptoms over the past 7 days				
* General practice physical activity questionnaire* (*GPPAQ*) [20]	✓	✓	✓	✓
• *Assesses physical activity levels (classified as active, moderately active, moderately inactive, inactive) in the past 7 days*				
* Scottish physical activity questionnaire* (*SPAQ*) [21]	✓	✓	✓	✓
• Captures 7-day recall of all leisure and occupational physical activity				
* Spinal cord injury exercise self-efficacy scale* (*ESES*) [22]	✓	✓	✓	✓
• Assesses current exercise self-efficacy				
* Brief fatigue inventory* (*BFI*) [23]	✓	✓	✓	✓
• Assesses severity of fatigue and any impact on functioning in the previous 24 hours				
* Eastern Cooperative Oncology Group* (*ECOG*) [24]	✓	✓	✓	✓
• Measures current performance status, how disease is progressing and affects daily living activities				
* Motivational ruler* [25]	✓	✓	✓	✓
• Includes two visual 100 mm visual analog scales to measure current importance and confidence in walking				
* Patient reported demographics and clinical history*	✓			
• Study specific measure includes sex, age, marital status, education, long-standing health conditions etc.				
* Adverse outcomes and events* (*intervention only*)		✓	✓	
* End of study questionnaire*				✓
• Effects on quality of life (intervention only); usefulness of information; achievement of physical activity goals; satisfaction with study/intervention; free text comments about the study				

^1^Randomization occurs after the baseline questionnaire has been returned. Therefore, follow-up questionnaire timings are as follows: *control group* - from the date of the post-randomization telephone call (for example, informing them they are in the control); *intervention group* - from the date of the motivational interview telephone call.

### Pedometers

Pedometers will be used to assess adherence to the walking intervention and objectively compare the amount walked by people in the intervention group with that walked in the control group. However, they are also an inexpensive and effective intervention to increase levels of physical activity [17]. In order to control for the impact of pedometers on walking behavior, only half of the participants in the intervention and control groups will be randomly allocated with pedometers. This is because wearing a pedometer might increase participants walking regardless of whether they are in the intervention or control group. They will be asked to wear them for seven consecutive days at each assessment point (baseline pedometer data will be provided once the research team has received a signed consent form) and to complete a form recording how many steps they took over the 7-day period.

### Primary outcome

The primary outcomes are whether participants find the study and intervention acceptable and whether a full randomized controlled trial is warranted and feasible.

### Secondary outcomes

The secondary outcomes are the refinement of the walking intervention based on views from participants and stakeholders and estimates of key aspects of trial design needed to design a full-scale randomized trial design, if warranted, including the following:

- The number of eligible participants, recruitment and retention rate, and response rates to initial and follow-up questionnaires
- The utility of the objective (pedometer) and subjective (self-report) methods to assess adherence to the walking intervention
- Estimates of the variability of the QoL

### Sample size

For the feasibility trial, we aim to consecutively recruit a minimum of 30 patients with recurrent or metastatic cancer to both the control and intervention groups (60 patients in total). A sample size of 30 per arm will be sufficient to estimate the standard deviation of our QoL outcomes and allow estimation of the true treatment difference. The latter is needed to perform a power calculation for the main study. A definitive sample size of a large-scale RCT will be determined from the results of this trial.

### Data management and analysis

All quantitative data will be double entered and subjected to basic descriptive statistical tests, including calculation of means and frequencies; 95% confidence intervals will be presented to display the imprecision in QoL measures. Independent *t*-tests and χ^2^ tests will be used for two-group measure comparisons. Their paired equivalents will be used to analyze changes in outcome measures between baseline and follow-up. Rates for process measures (for example, recruitment, attrition) will be calculated.

Reasons for non-participation and attrition will be collected to inform future recruitment and retention strategies. We will also collect data on age and gender, with consent, from all eligible people, regardless of whether they choose to participate or not, to explore whether participation rate varies according to these variables.

Audio recordings of interviews will be transcribed verbatim and analyzed using framework analysis; a widely used matrix-based method for collating, reviewing, and understanding qualitative data [26]. All data will be stored securely, and raw data will only be accessible to the study chief investigator (JA) and researchers (JH, VT).

### Serious adverse events reporting and monitoring

It is not anticipated that there will be any risk to participants. With permission, the researchers will write to all participants GPs to inform them of their participation on the study. Data on unexpected and serious adverse events will be collected at assessments T1 and T2. Any serious adverse events deemed to be related to the intervention or due to participation in the study will be reported to the chief investigator within 24 h of the team learning of its occurrence.

### Project management

The management of the feasibility study will be the responsibility of the research management group, comprising of the chief investigator, all co-applicants, and all research staff. Operational management will be the responsibility of the research team comprising of the chief investigator and research staff, meeting once a week to ensure adherence to planned timescale, adherence to the intervention and detailed plans for data management and analysis. An independent steering committee including health-care professionals, academic researchers, and service users will provide, oversee, and facilitate the study and will meet at least twice a year. This study was also adopted on to the UK National Cancer Research Network Portfolio (UKCRN ID 16236), which provides research infrastructure resources, including Clinical Trial Officer support for recruitment.

### Ethics and dissemination

This study has received ethical approval from the National Research Ethics Service (NRES) Committee North West - Lancaster and Health Research Authority NRES Centre - Manchester, and research and development governance approval from two London National Health Service (NHS) hospital trusts. Any planned changes to the study design will be submitted for further approval by NRES and relevant research and development committees. We will present study findings at conferences and publish them in peer-reviewed journals. Only individuals who fulfill the authorship criteria will be included as authors on final publications.

## Discussion

This study will explore the feasibility and acceptability of a community-based walking program in enhancing QoL outcomes in people with recurrent or metastatic cancer. To date, this is the first study investigating whether a brief walking intervention on its own is sufficient to enhance the health and well-being of these people. This is particularly important because although the number of people living with cancer continues to increase [2] people with recurrent or metastatic disease report reduced QoL [1].

Despite being the first of its kind, the intervention will only be available in English and therefore the uptake/views of minority ethnic groups may be underrepresented. Also, eight tumor types are included in the trial sample, which means that findings cannot be generalized to all tumor groups.

Findings from this study will determine whether recruitment to a full-scale trial is feasible and provide initial data for future sample size calculations. It will collect information on the acceptability of the walking intervention and permit refinement where necessary. Furthermore, the study will assess whether the outcomes being used are acceptable, appropriate and sensitive to change in this population. Finally, the study will investigate the acceptability of the use of pedometers and their influence on walking behavior.

## Trial status

Recruiting was closed on the 30th November 2014.

## Abbreviations

QoL: quality of life
NHS: National Health Service
NRES: National Research Ethics Service
NICE: National Institute for Health and Clinical Excellence
